# Lixisenatide improves glycemic outcomes of Japanese patients with type 2 diabetes: a meta-analysis

**DOI:** 10.1186/s13098-016-0151-7

**Published:** 2016-06-01

**Authors:** Hiroaki Seino, Yukiko Onishi, Yusuke Naito, Mitsuhisa Komatsu

**Affiliations:** Seino Internal Medicine Clinic, Fukushima, 9638851 Japan; Division of Clinical Trials, Division of Diabetes and Metabolism, The Institute for Adult Diseases, Asahi Life Foundation, Tokyo, Japan; Medical Affairs, Sanofi K.K., Tokyo, Japan; Division of Diabetes, Endocrinology and Metabolism, Department of Internal Medicine, Shinshu University School of Medicine, Nagano, Japan

**Keywords:** GLP-1 receptor agonists, Lixisenatide, Type 2 diabetes

## Abstract

**Background:**

The GetGoal-L-Asia and -S trials were multi-center trials conducted in 4 and 16 countries, respectively including Japan that evaluated the efficacy and safety of lixisenatide add-on treatment vs. placebo among patients with type 2 diabetes. The aims of this study were to determine the efficacy and safety of lixisenatide add-on treatment among Japanese patient groups.

**Methods:**

All Japanese intent-to-treat patients with baseline and endpoint HbA1c measurements were included in the meta-analyses. Subgroup analyses were carried out for patients with low (<8 %) and high (≥8 %) baseline HbA1c levels, low (<25 kg/m^2^) and high (≥25 kg/m^2^) baseline body mass index (BMI), short (<10 years) and long (≥10 years) durations of diabetes, and for those <65 and ≥65 years of age.

**Results:**

The overall study population of Japanese type 2 diabetes patients included 143 patients (mean age: 59.0 years; 35 % female) treated with lixisenatide and 136 patients treated with placebo (mean age: 57.8 years; 32 % female). Among the subgroups, lixisenatide treatment vs. placebo was associated with greater change in HbA1c (Low HbA1c −0.80 %, p < 0.0001; High HbA1c −1.19 %, p < 0.0001; low BMI −0.88 %, p < 0.0001; high BMI −1.28 %, p < 0.0001; short diabetes duration −1.28 %, p < 0.0001; long diabetes duration −0.93 %, p < 0.0001; <65 years: −1.00 %, p < 0.0001; ≥65 years −1.24 %, p < 0.0001). Additionally, among the subgroups, lixisenatide treatment vs. placebo was associated with greater change in post-prandial glucose.

**Conclusions:**

For Japanese type 2 diabetes patients lixisenatide may be an efficacious and safe add-on therapy leading to improved glycemic outcomes.

*GetGoal-L-Asia* NCT01169779

*GetGoal-S* NCT00713830

## Background

Type 2 diabetes is a metabolic disease resulting from insulin resistance and progressive beta-cell dysfunction. Its prevalence has increased worldwide in recent decades, and in Japan has increased from 6.9 million in 1997 to 9.5 million in 2012 making it a priority for the Japanese Ministry of Health, Labour and Welfare [1–4]. Some studies have reported differences in the pathophysiology of type 2 diabetes between Japanese patients and Caucasian patients, with that of Japanese patients being more related to reduced insulin secreting capacity than insulin resistance [5, 6]. However, a recent study reported that the differences in insulin sensitivity and beta-cell function between Japanese and Caucasians are no longer significant when differences in body mass index (BMI) and adipose tissue distribution are taken into consideration [7]. A World Health Organization expert review concluded that compared with Caucasians the risk for type 2 diabetes is higher for Asians, including the Japanese, at lower BMIs [8].

Glucagon-like peptide-1 (GLP-1) receptor agonists, including exenatide, liraglutide, dulaglutide, and lixisenatide are efficacious for the treatment of type 2 diabetes [9–13]. In addition to having glycemic efficacy, this class of drugs has other advantages such as the promotion of satiety and weight loss [9]. The GetGoal-L-Asia (NCT01169779) and -S (NCT00713830) trials were multicenter, multi-country trials that evaluated the efficacy and safety of lixisenatide add-on treatment vs. placebo among patients with type 2 diabetes with both trials having the primary endpoint of change in HbA1c [10, 11]. The GetGoal-L-Asia trial included 154 patients treated with lixisenatide (10 μg for 1 week, 15 μg for 1 week, then 20 μg once daily) and 157 patients treated with placebo from Japan, the Republic of Korea, Taiwan, and the Philippines [10]. All patients were also treated with basal insulin (glargine, detemir, NPH, premix) and ~70 % were also using a sulfonylurea drug [10]. The mean change at trial endpoint (24 weeks) in HbA1c was −0.77 % for lixisenatide treated patients and +0.11 % for placebo treated patients (treatment difference −0.88 %, p < 0.0001) [10]. Lixisenatide was also associated with significant decreases vs. placebo in 7-point self-monitored plasma glucose level, fasting plasma glucose (FPG) level, and daily basal insulin dose [10]. The GetGoal-S trial included 570 patients treated with lixisenatide (10 μg for 1 week, 15 μg for 1 week, then 20 μg once daily) and 286 patients treated with placebo from 16 different countries [11]. All patients in the GetGoal-S trial were also treated with a sulfonylurea drug and ~85 % were also using metformin [11]. The mean change at trial endpoint (24 weeks) in HbA1c was −0.85 % for lixisenatide treated patients and −0.10 % for placebo treated patients (treatment difference: −0.74 %, p < 0.0001) [11]. Both GetGoal-L-Asia and GetGoal-S trials included patients from Japan [10, 11]. The objectives of this study were to conduct meta-analyses of GetGoal-L-Asia and -S trial data to determine the efficacy and safety of lixisenatide add-on treatment among specific Japanese type 2 diabetes patient groups, including those with low (<8 %) and high (≥8 %) baseline HbA1c levels, low (<25 kg/m^2^) and high (≥25 kg/m^2^) baseline BMI, short (<10 years) and long (≥10 years) durations of diabetes, and those <65 and ≥65 years of age.

## Methods

### Study populations

All modified intent-to-treat Japanese patients living in Japan from the GetGoal-L-Asia and -S trials with baseline and endpoint HbA1c measurements reported were included in the meta-analyses. Analyses were carried out for subgroups with low (<8 %) and high (≥8 %) baseline HbA1c levels, low (<25 kg/m^2^) and high (≥25 kg/m^2^) baseline BMI, short (<10 years) and long (≥10 years) durations of diabetes, and those <65 and ≥65 years of age.

### Ethics statement

The GetGoal-L-Asia and -S trials were approved by the local ethics committee or institutional review boards and complied with the Declaration of Helsinki and the International Conference on harmonization—good clinical practice guidelines and all applicable amendments [10, 11].

### Meta-analyses method

The efficacy and safety of lixisenatide add-on treatment vs. placebo for different Japanese patient subgroups were evaluated by performing meta-analyses on the data from the clinical trials. For each efficacy outcome, including changes in HbA1c, weight, FPG, and post-prandial glucose (PPG, standardized 2-h meal test), the mean changes in baseline to endpoint measurements for the lixisenatide and placebo treatment arms of each subgroup were used. Other outcomes evaluated during the trial periods included the likelihoods of symptomatic and severe hypoglycemia, the likelihood of achieving an endpoint HbA1c <7 %, and the likelihoods of achieving the following composite endpoints: an endpoint HbA1c <7 % and no weight gain, an endpoint HbA1c <7 % and no symptomatic hypoglycemia, and an endpoint HbA1c <7 % and no weight gain and no symptomatic hypoglycemia.

Meta-analysis outcomes were assessed using a random effects model. Weighted mean differences with 95 % confidence intervals (CI) were determined for continuous data using the inverse variance method. Mantel–Haenszel odds ratios for 95 % CI were determined for all dichotomous outcome data. Heterogeneity between trials was assessed by $\chi^{2}$ test. All meta-analyses were conducted using Review Manager (RevMan, version 5.1, Copenhagen: Cochrane Collaboration). A *p* value of 0.05 was used to determine the level of statistical significance.

### Other summary statistics

The summary statistics of patient clinical characteristics as well as efficacy and safety outcomes for each treatment arm within the study groups were determined. The treatment arms within each subgroup were compared to each other with p values calculated using a Chi square test or ANOVA test where appropriate. A p value of 0.05 was used to determine the level of statistical significance. All descriptive statistical analyses were carried out using SAS^®^ 9.3 (Cary, NC).

## Results

The overall study population of Japanese type 2 diabetes patients included 143 patients (mean age: 59.0 years; 35 % female) treated with lixisenatide and 136 patients treated with placebo (mean age: 57.8 years; 32 % female). The mean durations of type 2 diabetes were 11.9 and 12.4 years among patients treated with lixisenatide and placebo, respectively. Lixisenatide treated patients had significantly greater changes in HbA1c (−1.08 %, confidence interval (CI) −1.29, −0.86, p < 0.0001) and PPG levels (−149.8 mg/dL, CI −170.4, −129.2, p < 0.0001) in comparison to placebo treated patients during trial periods. Lixisenatide treated patients had a greater likelihood of having symptomatic hypoglycemia during the trial periods in comparison to placebo treated patients [odds ratio (OR) 3.0, CI 1.4, 6.3, p = 0.0040]; however, lixisenatide treated patients had greater likelihoods of achieving an endpoint HbA1c <7 % (OR 20.3, CI 6.1, 67.8, p < 0.0001), and the composite endpoints of an HbA1c <7 % and no weight gain (OR 13.5, CI 4.0, 45.6, p < 0.0001), an HbA1c < 7 % and no symptomatic hypoglycemia (OR 18.5, CI 4.3, 78.8, p < 0.0001), and an HbA1c <7 % and no weight gain and no symptomatic hypoglycemia (OR 12.8, CI 2.9, 55.7, p = 0.0007) in comparison to placebo treated patients. Severe hypoglycemia was not observed among the overall study population of Japanese type 2 diabetes patients.

**Fig. 1:**
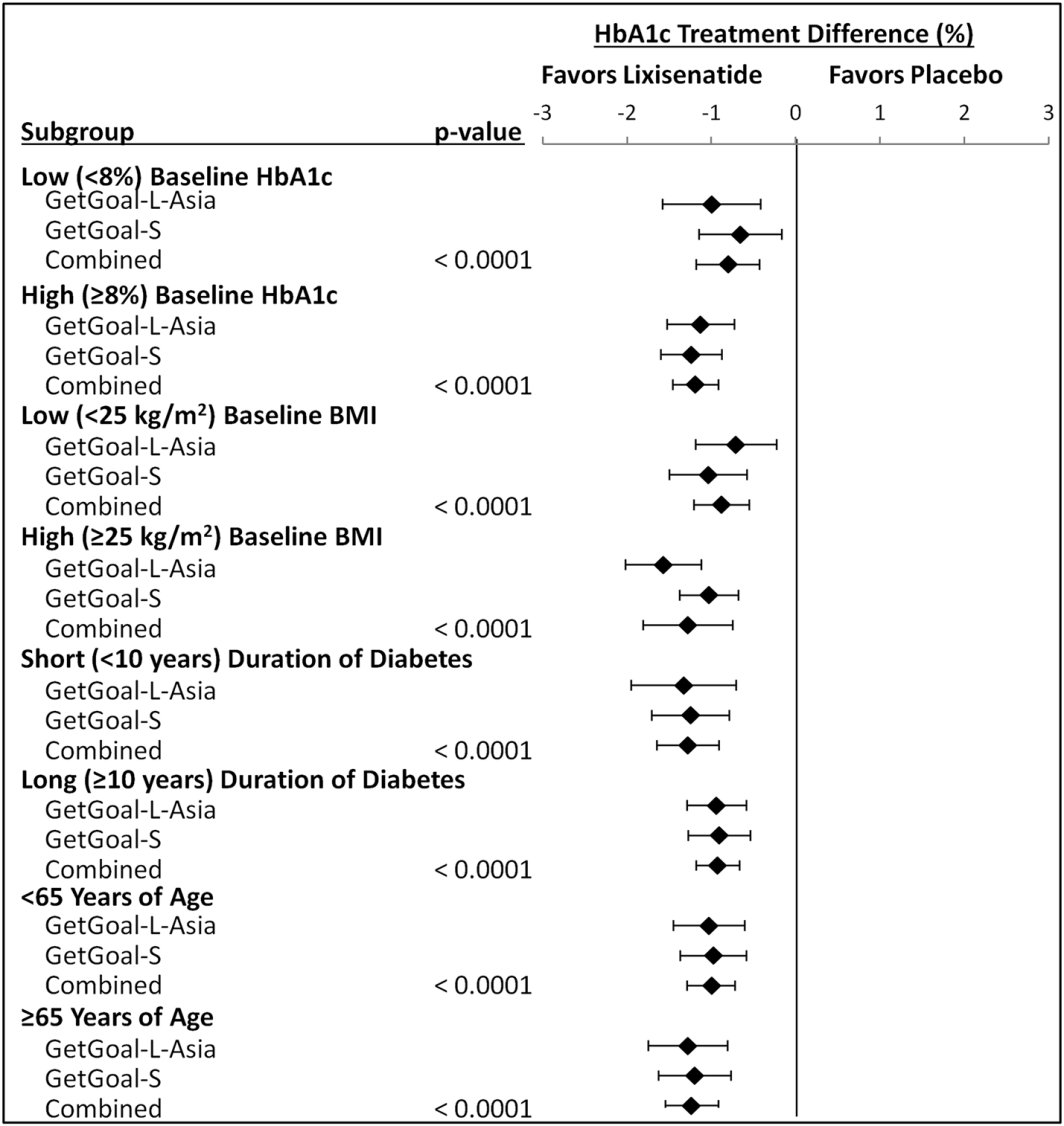
*Forest plot* of hba1c treatment differences among Japanese type 2 diabetes patient subgroups

Baseline characteristics of study subgroups are presented in Table 1. Figure 1 presents a forest plot of mean treatment differences of changes in HbA1c during trial periods of Japanese type 2 diabetes patient subgroups.

**Table 1:** Baseline characteristics of study subgroups

	Lixisenatide	Placebo	p value	Lixisenatide	Placebo	p value
	Low (<8 %) baseline HbA1c			High (≥8 %) baseline HbA1c		
N	44	33		99	103	
Age (years), mean (SD)	61.2 (9.1)	58.7 (11.3)	0.29	58.0 (10.9)	57.5 (11.3)	0.75
Female-N (%)	14 (31.8)	8 (24.2)	0.47	36 (36.4)	36 (35.0)	0.83
Duration of diabetes (years), mean (SD)	13.1 (8.0)	12.8 (10.0)	0.89	11.3 (7.3)	12.3 (7.4)	0.34
HbA1c (%), mean (SD)	7.53 (0.29)	7.52 (0.29)	0.86	8.88 (0.62)	8.90 (0.58)	0.81

	Low (<25 kg/m^2^) baseline BMI			High (≥25 kg/m^2^) baseline BMI		
N	77	73		66	63	
Age (years), mean (SD)	60.9 (8.9)	59.8 (10.9)	0.49	56.7 (11.7)	55.4 (11.4)	0.53
Female-N (%)	21 (27.3)	24 (32.9)	0.45	29 (43.9)	20 (31.8)	0.15
Duration of diabetes (years), mean, (SD)	12.4 (7.5)	14.1 (9.1)	0.22	11.2 (7.5)	10.5 (6.2)	0.56
HbA1c (%), mean (SD)	8.38 (0.81)	8.41 (0.73)	0.79	8.56 (0.84)	8.74 (0.84)	0.23

	Short (<10 years) duration of diabetes			Long (≥10 years) duration of diabetes		
N	68	59		75	77	
Age (years), mean (SD)	55.9 (11.3)	53.3 (10.4)	0.19	61.8 (8.9)	61.2 (10.8)	0.71
Female-N (%)	27 (39.7)	16 (27.1)	0.13	23 (30.7)	28 (36.4)	0.46
Duration of diabetes (years), mean (SD)	5.5 (2.8)	5.9 (2.2)	0.42	17.7 (5.5)	17.5 (7.2)	0.86
HbA1c (%), mean (SD)	8.52 (0.81)	8.64 (0.86)	0.43	8.41 (0.84)	8.50 (0.74)	0.46

	<65 years of age			≥65 years of age		
N	97	97		46	39	
Age (years), mean (SD)	53.9 (8.6)	52.6 (8.8)	0.30	69.7 (3.9)	70.7 (4.2)	0.25
Female-N (%)	35 (36.1)	30 (30.9)	0.45	15 (32.6)	14 (35.9)	0.75
Duration of diabetes (years), mean, (SD)	10.4 (6.9)	10.5 (6.5)	0.92	14.9 (8.0)	17.2 (9.6)	0.24
HbA1c (%), mean (SD)	8.56 (0.83)	8.82 (0.78)	0.16	8.24 (0.87)	8.44 (0.74)	0.25

*SD* standard deviation; *BMI* body mass index

### Subgroups: Japanese type 2 diabetes patients with low (<8 %) and high (≥8 %) baseline HbA1c levels

A summary of the meta-analysis outcomes of Japanese type 2 diabetes patients with low (<8 %) and high (≥8 %) baseline HbA1c levels placebo is presented in Table 2. Lixisenatide treated patients with low (<8 %) baseline HbA1c had significantly greater changes in HbA1c (−0.80 %, CI −1.18, −0.43, p < 0.0001) and PPG levels (−132.0 mg/dL, CI −171.7, −92.4, p < 0.0001) in comparison to placebo treated patients with low (<8 %) baseline HbA1c during trial periods. The likelihood of symptomatic hypoglycemia occurring during trial periods was not significantly different among patients with low (<8 %) baseline HbA1c treated with lixisenatide and placebo. Lixisenatide treated patients with low (<8 %) baseline HbA1c had greater likelihoods of achieving an endpoint HbA1c <7 % (OR 25.5, CI 5.2, 124.6, p < 0.0001), and the composite endpoints of an HbA1c <7 % and no weight gain (OR 15.8, CI 3.3, 75.8, p = 0.0005), an HbA1c <7 % and no symptomatic hypoglycemia (OR 11.8, CI 2.0, 68.7, p = 0.0060), and an HbA1c <7 % and no weight gain and no symptomatic hypoglycemia (OR 8.9, CI 1.5, 52.2, p = 0.0154).

**Table 2:** Summary of meta-analysis outcomes for Japanese type 2 diabetes patients with low (<8 %) and high (≥8 %) baseline HbA1c levels

Outcome	Effect estimate	95 % Confidence limits		p value	Effect estimate	95 % Confidence limits		p value
		Lower	Upper			Lower	Upper	
		Low (<8 %) baseline HbA1c				High (≥8 %) baseline HbA1c		
Change in HbA1c (%)	−0.80	−1.18	−0.43	<0.0001	−1.19	−1.46	−0.92	<0.0001
Change in weight (kg)	−0.62	−1.72	0.49	0.28	−0.17	−0.68	0.34	0.52
Change in FPG (mg/dL)	−12.5	−30.9	5.8	0.18	−14.7	−25.9	−3.6	0.0097
Change in PPG: meal test (mg/dL)	−132.0	−171.7	−92.4	<0.0001	−162.0	−185.9	−138.1	<0.0001

	Odds ratio	95 % Confidence limits		p value	Odds ratio	95 % Confidence limits		p value
		Lower	Upper			Lower	Upper	
		Low (<8 %) baseline HbA1c				High (≥8 %) baseline HbA1c		
Symptomatic hypoglycemia	2.9	0.8	10.0	0.10	2.9	1.2	7.1	0.0229
Severe hypoglycemia	0.0	0.0	0.0	–	0.0	0.0	0.0	–
Endpoint HbA1c <7 %	25.5	5.2	124.6	<0.0001	15.8	2.9	85.6	0.0014
Endpoint HbA1c <7 % and no weight gain	15.8	3.3	75.8	0.0005	10.0	1.8	55.9	0.0091
Endpoint HbA1c <7 % and no symptomatic hypoglycemia	11.8	2.0	68.7	0.0060	11.1	2.0	61.8	0.0059
Endpoint HbA1c <7 % and no weight gain and no symptomatic hypoglycemia	8.9	1.5	52.2	0.0154	6.4	1.1	38.6	0.0430

*FPG* fasting plasma glucose, *PPG* postprandial glucose

Lixisenatide treated patients with high (≥8 %) baseline HbA1c had significantly greater changes in HbA1c (−1.19 %, CI −1.46, −0.92, p < 0.0001), FPG (−14.7 mg/dL, CI −25.9, −3.6, p = 0.0097), and PPG levels (−162.0 mg/dL, CI −185.9, −138.1, p < 0.0001) in comparison to placebo treated patients with high (≥8 %) baseline HbA1c during trial periods. Lixisenatide treated patients with high (≥8 %) baseline HbA1c had a greater likelihood of having symptomatic hypoglycemia during the trial periods in comparison to placebo treated patients (OR 2.9, CI 1.2, 7.1, p = 0.0229); however, lixisenatide treated patients had greater likelihoods of achieving an endpoint HbA1c <7 % (OR 15.8, CI 2.9, 85.6, p = 0.0014), and the composite endpoints of an HbA1c <7 % and no weight gain (OR 10.0, CI 1.8, 55.9, p = 0.0091), an HbA1c <7 % and no symptomatic hypoglycemia (OR 11.1, CI 2.0, 61.8, p = 0.0059), and an HbA1c <7 % and no weight gain and no symptomatic hypoglycemia (OR 6.4, CI 1.1, 38.6, p = 0.0430) in comparison to placebo treated patients with high (≥8 %) baseline HbA1c.

### Subgroups: Japanese type 2 diabetes patients with low (<25 kg/m^2^) and high (≥25 kg/m^2^) baseline BMI

A summary of the meta-analysis outcomes of Japanese type 2 diabetes patients with low (<25 kg/m^2^) and high (≥25 kg/m^2^) baseline BMI is presented in Table 3. Lixisenatide treated patients with low (< 25 kg/m^2^) baseline BMI had significantly greater changes in HbA1c (−0.88 %, CI −1.21, −0.55, p < 0.0001) and PPG levels (−147.9 mg/dL, CI −178.6, −117.3, p < 0.0001) in comparison to placebo treated patients with low (<25 kg/m^2^) baseline BMI during trial periods. Lixisenatide treated patients with low (<25 kg/m^2^) baseline BMI had a greater likelihood of having symptomatic hypoglycemia during the trial periods in comparison to placebo treated patients (OR 2.8, CI 1.1, 7.0, p = 0.0270); however, lixisenatide treated patients had greater likelihoods of achieving an endpoint HbA1c <7 % (OR 11.2, CI 3.2, 39.7, p = 0.0002), and the composite endpoints of an HbA1c <7 % and no weight gain (OR 7.6, CI 2.1, 27.9, p = 0.0024), an HbA1c <7 % and no symptomatic hypoglycemia (OR 9.6, CI 2.1, 43.7, p = 0.0036), and an HbA1c <7 % and no weight gain and no symptomatic hypoglycemia (OR 7.4, CI 1.6, 35.2, p = 0.0114) in comparison to placebo treated patients with low (<25 kg/m^2^) baseline BMI.

**Table 3:** Summary of meta-analysis outcomes for Japanese type 2 diabetes patients with low (<25 kg/m^2^) and high (≥25 kg/m^2^) baseline body mass index (BMI)

Outcome	Effect estimate	95 % Confidence limits		p value	Effect estimate	95 % Confidence limits		p value
		Lower	Upper			Lower	Upper	
		Low (<25 kg/m^2^) baseline BMI				High (≥25 kg/m^2^) baseline BMI		
Change in HbA1c (%)	−0.88	−1.21	−0.55	<0.0001	−1.28	−1.81	−0.75	<0.0001
Change in weight (kg)	−0.38	−1.07	0.32	0.29	−0.28	−1.18	0.61	0.53
Change in FPG (mg/dL)	−5.9	−19.1	7.5	0.39	−21.2	−45.8	3.5	0.09
Change in PPG: meal: test (mg/dL)	−147.9	−178.6	−117.3	<0.0001	−151.5	−178.8	−124.3	<0.0001

	Odds ratio	95 % Confidence limits		p value	Odds ratio	95 % Confidence limits		p value
		Lower	Upper			Lower	Upper	
		Low (<25 kg/m^2^) baseline BMI				High (≥25 kg/m^2^) baseline BMI		
Symptomatic hypoglycemia	2.8	1.1	7.0	0.0270	4.2	1.1	15.7	0.0362
Severe hypoglycemia	0.0	0.0	0.0	–	0.0	0.0	0.0	–
Endpoint HbA1c <7 %	11.2	3.2	39.7	0.0002	23.6	3.0	183.6	0.0025
Endpoint HbA1c <7 % and no weight gain	7.6	2.1	27.9	0.0024	16.2	2.1	127.2	0.0083
Endpoint HbA1c <7 % and no symptomatic hypoglycemia	9.6	2.1	43.7	0.0036	15.6	2.0	123.7	0.0093
Endpoint HbA1c <7 % and no weight gain and no symptomatic hypoglycemia	7.4	1.6	32.2	0.0114	9.5	1.2	77.7	0.0356

*FPG* fasting plasma glucose, *PPG* postprandial glucose

Lixisenatide treated patients with high (≥25 kg/m^2^) baseline BMI had significantly greater changes in HbA1c (−1.28 %, CI −1.81, −0.75, p < 0.0001) and PPG levels (−151.5 mg/dL, CI −178.8, −124.3, p < 0.0001) in comparison to placebo treated patients with high (≥25 kg/m^2^) baseline BMI during trial periods. Lixisenatide treated patients with high (≥25 kg/m^2^) baseline BMI had a greater likelihood of having symptomatic hypoglycemia during the trial periods in comparison to placebo treated patients (OR 4.2, CI 1.1, 15.7, p = 0.0362); however, lixisenatide treated patients had greater likelihoods of achieving an endpoint HbA1c <7 % (OR 23.6, CI 3.0, 183.6, p = 0.0025), and the composite endpoints of an HbA1c <7 % and no weight gain (OR 16.2, CI 2.1, 127.2, p = 0.0083), an HbA1c <7 % and no symptomatic hypoglycemia (OR 15.6, CI 2.0, 123.7, p = 0.0093), and an HbA1c <7 % and no weight gain and no symptomatic hypoglycemia (OR 9.5, CI 1.2, 77.7, p = 0.0356) in comparison to placebo treated patients with high (≥25 kg/m^2^) baseline BMI.

### Subgroups: Japanese type 2 diabetes patients with short (<10 years) and long (≥10 years) durations of diabetes

A summary of the meta-analysis outcomes of Japanese type 2 diabetes patients with short (<10 years) and long (≥10 years) durations of diabetes is presented in Table 4. Lixisenatide treated patients with short (<10 years) duration of diabetes had significantly greater changes in HbA1c (−1.28 %, CI −1.65, −0.91, p < 0.0001) and PPG levels (−152.6 mg/dL, CI −182.0, −123.2, p < 0.0001) in comparison to placebo treated patients with short (<10 years) duration of diabetes. Lixisenatide treated patients with short (<10 years) duration of diabetes had a greater likelihood of having symptomatic hypoglycemia during the trial periods in comparison to placebo treated patients (OR 5.2, CI 1.5, 18.7, p = 0.0107); however, lixisenatide treated patients had greater likelihoods of achieving an endpoint HbA1c <7 % (OR 32.1, CI 4.2, 246.6, p = 0.0009), and the composite endpoints of an HbA1c <7 % and no weight gain (OR 22.5, CI 2.9, 174.3, p = 0.0028), an HbA1c <7 % and no symptomatic hypoglycemia (OR 19.8, CI 2.6, 153.5, p = 0.0043), and an HbA1c <7 % and no weight gain and no symptomatic hypoglycemia (OR 11.6, CI 1.4, 93.3, p = 0.0213) in comparison to placebo treated patients with short (<10 years) duration of diabetes.

**Table 4:** Summary of meta-analysis outcomes for Japanese type 2 diabetes patients with short (<10 years) and long (≥10 years) duration of diabetes

Outcome	Effect estimate	95 % Confidence limits		p value	Effect estimate	95 % Confidence limits		p value
		Lower	Upper			Lower	Upper	
		Short (<10 years) duration of diabetes				Long (≥10 years) duration of diabetes		
Change in HbA1c (%)	−1.28	−1.65	−0.91	<0.0001	−0.93	−1.18	−0.67	<0.0001
Change in weight (kg)	−0.56	−1.34	0.21	0.16	−0.09	−0.66	0.47	0.75
Change in FPG (mg/dL)	−12.7	−26.6	1.2	0.07	−12.6	−24.9	−0.4	0.0438
Change in PPG: meal test (mg/dL)	−152.6	−182.0	−123.2	<0.0001	−147.9	−176.8	−119.0	<0.0001

	Odds ratio	95 % Confidence limits		p value	Odds ratio	95 % Confidence limits		p value
		Lower	Upper			Lower	Upper	
		Short (<10 years) duration of diabetes				Long (≥10 years) duration of diabetes		
Symptomatic hypoglycemia	5.2	1.5	18.7	0.0107	2.1	0.8	5.2	0.11
Severe hypoglycemia	0.0	0.0	0.0	–	0.0	0.0	0.0	–
Endpoint HbA1c <7 %	32.1	4.2	246.6	0.0009	8.9	2.5	31.9	0.0008
Endpoint HbA1c <7 % and no weight gain	22.5	2.9	174.3	0.0028	6.3	1.7	23.3	0.0057
Endpoint HbA1c <7 % and no symptomatic hypoglycemia	19.8	2.6	153.5	0.0043	9.0	2.0	41.1	0.0046
Endpoint HbA1c <7 % and no weight gain and no symptomatic hypoglycemia	11.6	1.4	93.3	0.0213	7.5	1.6	34.7	0.0099

*FPG* fasting plasma glucose, *PPG* postprandial glucose

Lixisenatide treated patients with a long (≥10 years) duration of diabetes had significantly greater changes in HbA1c (−0.93 %, CI −1.18, −0.67, p < 0.0001), FPG (−12.6 mg/dL, CI −24.9, −0.4, p = 0.0438), and PPG levels (−147.9 mg/dL, CI −176.8, −119.0, p < 0.0001) in comparison to placebo treated patients with long (≥10 years) duration of diabetes. Lixisenatide treated patients with long (≥10 years) duration of diabetes had greater likelihoods of achieving an endpoint HbA1c <7 % (OR 8.9, CI 2.5, 31.9, p = 0.0008), and the composite endpoints of an HbA1c <7 % and no weight gain (OR 6.3, CI 1.7, 23.3, p = 0.0057), an HbA1c <7 % and no symptomatic hypoglycemia (OR 9.0, CI 2.0, 41.1, p = 0.0046), and an HbA1c <7 % and no weight gain and no symptomatic hypoglycemia (OR 7.5, CI 1.6, 34.7, p = 0.0099) in comparison to placebo treated patients with long (≥10 years) duration of diabetes.

### Subgroups: Japanese type 2 diabetes patients <65 and ≥65 years of age

A summary of the meta-analysis outcomes of Japanese type 2 diabetes patients <65 and ≥65 years of age is presented in Table 5. Lixisenatide treated patients <65 years of age had significantly greater changes in HbA1c (−1.00 %, CI −1.29, −0.72, p < 0.0001) and PPG levels (−138.1 mg/dL, CI −177.0, −99.2, p < 0.0001) in comparison to placebo treated patients <65 years of age. The likelihood of symptomatic hypoglycemia occurring during trial periods trended to be greater for patients <65 years of age treated with lixisenatide than placebo, but did not reach significance (OR 2.3, CI 1.0, 5.6, p = 0.06). Lixisenatide treated patients <65 years of age had greater likelihoods of achieving an endpoint HbA1c <7 % (OR 13.1, CI 3.4, 50.7, p = 0.0002), and the composite endpoints of an HbA1c <7 % and no weight gain (OR 8.4, CI 2.1, 33.8, p = 0.0026), an HbA1c <7 % and no symptomatic hypoglycemia (OR 14.0, CI 2.6, 76.8, p = 0.0023), and an HbA1c <7 % and no weight gain and no symptomatic hypoglycemia (OR 7.8, CI 1.3, 45.6, p = 0.0221) in comparison to placebo treated patients <65 years of age.

**Table 5:** Summary of meta-analysis outcomes for Japanese type 2 diabetes patients <65 and ≥65 years of age

Outcome	Effect estimate	95 % Confidence limits		p value	Effect estimate	95 % Confidence limits		p value
		Lower	Upper			Lower	Upper	
		<65 years of age				≥65 years of age		
Change in HbA1c (%)	−1.00	−1.29	−0.72	<0.0001	−1.24	−1.55	−0.92	<0.0001
Change in weight (kg)	−0.15	−1.07	0.76	0.74	−0.58	−1.39	0.22	0.16
Change in FPG (mg/dL)	−11.2	−23.3	0.9	0.07	−14.8	−29.9	0.3	0.06
Change in PPG: meal test (mg/dL)	−138.1	−177.0	−99.2	<0.0001	−166.5	−228.3	−104.6	<0.0001

	Odds ratio	95 % Confidence limits		p value	Odds Ratio	95 % Confidence limits		p value
		Lower	Upper			Lower	Upper	
		<65 years of age				≥65 years of age		
Symptomatic hypoglycemia	2.3	1.0	5.6	0.06	5.1	1.3	19.7	0.0197
Severe hypoglycemia	0.0	0.0	0.0	–	0.0	0.0	0.0	–
Endpoint HbA1c <7 %	13.1	3.4	50.7	0.0002	23.2	4.1	131.9	0.0004
Endpoint HbA1c <7 % and no weight gain	8.4	2.1	33.8	0.0026	16.7	2.9	95.7	0.0015
Endpoint HbA1c <7 % and no symptomatic hypoglycemia	14.0	2.6	76.8	0.0023	12.3	2.1	70.7	0.0049
Endpoint HbA1c <7 % and no weight gain and no symptomatic hypoglycemia	7.8	1.3	45.6	0.0221	10.1	1.7	58.3	0.0100

*FPG* fasting plasma glucose, *PPG* postprandial glucose

Lixisenatide treated patients ≥65 years of age had significantly greater changes in HbA1c (−1.24 %, CI −1.55, −0.92, p < 0.0001) and PPG levels (−166.5 mg/dL, CI −228.3, −104.6, p < 0.0001) in comparison to placebo treated patients ≥65 years of age. Lixisenatide treated patients ≥65 years of age had a greater likelihood of having symptomatic hypoglycemia during the trial periods in comparison to placebo treated patients (OR 5.1, CI 1.3, 19.7, p = 0.0197); however, lixisenatide treated patients had greater likelihoods of achieving an endpoint HbA1c <7 % (OR 23.2, CI 4.1, 131.9, p = 0.0004), and the composite endpoints of an HbA1c <7 % and no weight gain (OR 16.7, CI 2.9, 95.7, p = 0.0015), an HbA1c <7 % and no symptomatic hypoglycemia (OR 12.3, CI 2.1, 70.7, p = 0.0049), and an HbA1c <7 % and no weight gain and no symptomatic hypoglycemia (OR 10.1, CI 1.7, 58.3, p = 0.0100) in comparison to placebo treated patients ≥65 years of age.

## Discussion

The findings of the meta-analyses of the GetGoal-L-Asia and -S trials showed that among all Japanese type 2 diabetes patient subgroups examined lixisenatide add-on treatment vs. placebo was associated with significant reductions in HbA1c and PPG levels during the trial periods. The estimated differences in the change in HbA1c between lixisenatide and placebo treatment arms across the Japanese patient subgroups ranged from −0.80 to −1.28 %. The changes in HbA1c among the subgroups were similar to that observed for the overall Japanese populations of the GetGoal-L-Asia and -S trials (GetGoal-L-Asia: −1.1 %; GetGoal-S: −1.1 %) [14, 15]. The estimated differences in PPG levels between lixisenatide and placebo treatment arms across the subgroups ranged from −132.0 to −166.5 mg/dL and were also similar to that of the overall Japanese populations of the GetGoal-L-Asia and -S trials (GetGoal-L-Asia: −155.7 mg/dL; GetGoal-S: −153.3 mg/dL) [14, 15]. The impact of lixisenatide add-on treatment among Japanese patients with type 2 diabetes was greater than that reported in a meta-analysis of 14 randomized control trials that included patients with diabetes from multiple countries [16]. This meta-analysis reported that compared to placebo, lixisenatide significantly reduced HbA1c by −0.52 % and PPG level by −82 mg/dL [16]. The effect of lixisenatide treatment on PPG levels was similar to that observed among Japanese patients in the short-term PDY6797 study (−160.3 mg/dL) [17]. FPG levels were only significantly reduced among lixisenatide vs. placebo treated Japanese type 2 diabetes patients who had a baseline HbA1c ≥8 % or a ≥10 year duration of diabetes; however, there was a trend for FPG reduction in all other lixisenatide treated arms of subgroups.

As the effects of lixisenatide treatment on FPG levels were modest among the subgroups of Japanese type 2 diabetes patients it appears the much greater impact of lixisenatide add-on treatment on PPG levels provides the predominate benefit to greater HbA1c control. Wang et al. (2011) did report that the contribution of PPG to excess hyperglycemia is greater than FPG in well-controlled Asian type 2 diabetes patients and equally important as FPG in moderately to poorly controlled Asian type 2 diabetes patients [18]. The substantial lowering of PPG level associated with lixisenatide treatment may also potentially provide a cardiovascular benefit among Japanese type 2 diabetes patients [15, 17]. A long-term evaluation of the Kumamoto study, which included 110 Japanese type 2 diabetes patients found that intensive glycemic control, which included a threshold of a 2-h PPG <180 mg/dL, along with thresholds of FPG <110 mg/dL and HbA1c <6.5 % can delay the onset and progression of microvascular complications [19]. Some studies have shown that high PPG, independent of FPG, is associated with cardiovascular morbidity and mortality among patients with type 2 diabetes, although others, notably interventional clinical trials, are conflicting [20, 21]. A recently published study of 775 Japanese Americans did find that temporary hyperglycemia was correlated with oxidative stress, which likely plays a role in diabetic vascular complications [22, 23]. Additionally, a cross-sectional study of Chinese type 2 diabetes patients using continuous glucose monitoring demonstrated glycemic variability is associated with subclinical atherosclerosis [24]. The influence of lixisenatide on macrovascular complications among patients with type 2 diabetes and acute coronary syndrome was recently evaluated in the multicenter ELIXA study (Evaluation of Cardiovascular Outcomes in Patients with Type 2 Diabetes after Acute Coronary Syndrome During Treatment with AVE0010 [Lixisenatide], NCT01147250), which reported that the risk for major cardiovascular events was not significantly impacted by adding lixisenatide to usual care [25]. As optimal glycemic control has been shown to have a more pronounced effect on reducing the risk for microvascular complications among patients with type 2 diabetes it will be important in future studies to also examine whether lixisenatide treatment is associated with reduced risk for the development of microvascular complications [26].

Lixisenatide add-on treatment vs. placebo was associated with a greater likelihood of symptomatic hypoglycemia during trial periods for Japanese type 2 diabetes patients who had a baseline HbA1c ≥8 %, or a low or high baseline BMI, or a diabetes duration <10 years, or who were ≥65 years of age. However, among all subgroups evaluated lixisenatide add-on treatment vs. placebo was associated with significantly greater odds for achieving an HbA1c <7 % at the end of the trial periods and achieving the glycemic target without symptomatic hypoglycemia. There were no severe hypoglycemia events observed among the Japanese populations of these two trials indicating that the hypoglycemia differences were related to non-severe hypoglycemia frequency.

Among the overall Japanese GetGoal-L-Asia and -S trial populations lixisenatide treatment was associated with a beneficial effect on body weight, with mean reductions of −0.85 kg and −1.12 kg respectively [14, 15]. In our meta-analysis of the subgroups of Japanese type 2 diabetes patients from these two trials lixisenatide treatment also tended to be associated with weight loss, although the reductions were not statistically significant vs. placebo. In the GetGoal-L- Asia and -S trials type 2 diabetes patients were treated with basal insulin and/or sulfonylurea, both of which are associated with weight gain [10, 11]. Thus, our results are consistent with the theory that lixisenatide treatment mitigates weight gain caused by basal insulin and/or sulfonylurea treatment. Furthermore, lixisenatide vs. placebo treatment was associated with a much greater likelihood of achieving an endpoint HbA1c <7 % without weight gain during the trial periods across all subgroups evaluated (odds ratios: 6.3–22.5). Japanese patients with type 2 diabetes and ≥65 years of age treated with lixisenatide vs. placebo had a 16.7-fold greater likelihood for having an endpoint HbA1c <7 % and no weight gain. As type 2 diabetes is a risk factor for sarcopenia/frailty (i.e., progressive loss of muscle mass and strength) and prevalent among elderly persons, good glycemic control along with weight loss may prevent its progression [27, 28].

This meta-analyses was conducted on specific subgroups from a limited number of trials, which potentially may have led to reduced statistical power to detect differences in treatment arms of subgroups. Although the intent of this study was to specifically evaluate the effects of lixisenatide add-on treatment among Japanese type 2 diabetes patients, this also made the results less applicable to other type 2 diabetes patient populations. In the GetGoal-L- Asia and -S trials type 2 diabetes patients were treated with different anti-diabetic drugs (i.e., different basal insulins, metformin, sulfonylureas) at different dosages in combination with lixisenatide or placebo [10, 11]. Differences in combinations and dosages of anti-diabetic medications may influence the outcomes, such as weight change, of the two trial populations. However, the randomized nature of the design of these trials may partially ameliorate such differences between the trial arms of each respective trial [10, 11]. Furthermore, also largely explained by randomization of the trial populations, are the generally similar baseline HbA1c values, FPG levels, and PPG levels of the trials arms of the GetGoal-L-Asia and -S trials [10, 11]. For example, baseline HbA1c values of type 2 diabetes patients in the GetGoal-L-Asia trial were 8.54 and 8.52 % for patients in the lixisenatide and placebo add-on trials arms, respectively [10]. This was also true among type 2 diabetes patients treated with lixisenatide and placebo add-on treatment in the GetGoal-S trial, which were 8.3 and 8.2 %, respectively [11].

For this study we examined several patient subgroups to better understand the utility of lixisenatide add-on treatment for improving glycemic control among Japanese type 2 diabetes patients with different characteristics. Although, the results of the meta-analyses show that regardless of baseline HbA1c level, baseline BMI, duration of diabetes, or age, add-on of lixisenatide treatment was associated with improvement in glycemic control and other outcomes, there were differences in the size of the effect. Additionally, other factors than evaluated in our meta-analyses, such as baseline FPG and PPG levels, may also influence response to treatment with anti-diabetic medications and further study is warranted. The interaction of certain characteristics of type 2 diabetes patients, like that of BMI and HbA1c level, may also influence responses to treatments and outcomes of type 2 diabetes patients. Further research is needed to evaluate the potential interactions of such factors, and how these outcomes might be translated into the real-world of Japanese patients with type 2 diabetes. Moreover, it will be important to study patients with certain characteristics for longer periods of time in the real-world setting, especially in regard to the risk for developing microvascular and macrovascular complications. Lastly, the results of the clinical trial meta-analysis are limited by the patient types and trial designs as evaluated in the GetGoal-L-Asia and -S trials, of which the former did not include reporting of detailed data on outcomes, such as changes in blood pressure. Additionally, this meta-analysis did not evaluate other adverse events, except hypoglycemia.

## Conclusion

Among Japanese type 2 diabetes patients, regardless of HbA1c level, BMI, duration of diabetes, or age, lixisenatide may be an efficacious and a safe add-on therapy, when hypoglycemia risk is taken into consideration, leading to improved glycemic outcomes.

## Abbreviations

BMI: body mass index
GLP-1: glucagon-like peptide-1
HbA1c: glycated hemoglobin
FPG: fasting plasma glucose
PPG: post prandial glucose
